# Brain MRI Revealing the Probable Pathophysiology of Neuropsychiatric Lupus: A Case Report

**DOI:** 10.7759/cureus.90993

**Published:** 2025-08-25

**Authors:** Giuseppina S Simone, Jake H Gordon, Amrita Ravi, Ning Zhong, Forshing Lui

**Affiliations:** 1 Internal Medicine, California Northstate University College of Medicine, Elk Grove, USA; 2 Emergency Medicine, California Northstate University College of Medicine, Elk Grove, USA; 3 Psychiatry, California Northstate University College of Medicine, Elk Grove, USA; 4 Neurology, California Northstate University College of Medicine, Elk Grove, USA; 5 Neurology, Kaiser Permanente Sacramento Medical Center, Sacramento, USA

**Keywords:** autoimmune limbic encephalitis, clinical case report, cns lupus, neuropsychiatric sle, pathophysiology

## Abstract

Systemic lupus erythematosus (SLE) is a chronic systemic autoimmune disease with a wide array of manifestations in different organ systems. Involvement of the central nervous system (CNS), also known as neuropsychiatric SLE (NPSLE), often presents with seizures, mental status changes, focal neurological findings, and cognitive impairment. While many underlying pathophysiologic mechanisms have been described, no unifying etiology has been determined for NPSLE, thus complicating treatment.

We highlight the case of a 41-year-old female with SLE who presented to the emergency department after several generalized tonic-clonic seizure episodes. On physical examination, she was oriented only to self, had poor speech production, and responded only to noxious stimuli. Electroencephalography (EEG) showed right temporal focal slowing followed by a right temporal focal onset seizure. The patient was started on levetiracetam, lacosamide, and valproic acid. Pertinent lab results showed active systemic lupus. Her brain MRI revealed relatively symmetric bilateral medial temporal fluid-attenuated inversion recovery (FLAIR) hyperintensities compatible with limbic encephalitis. Her neurological condition and seizures persisted despite treatment with multiple anticonvulsants and oral prednisone. Once intravenous immunoglobulin (IVIg) was initiated, rapid symptom relief was achieved within 48 hours in refractory NPSLE with limbic encephalitis features, highlighting its efficacy when anticonvulsants (levetiracetam, lacosamide, valproic acid) and steroids (prednisone 60 mg) fail.

This case demonstrates the importance of understanding the various underlying pathophysiologies of NPSLE to better target clinical management. The use of modern neuroimaging MRI techniques may aid in this pursuit.

## Introduction

Systemic lupus erythematosus (SLE), a chronic systemic autoimmune disease, is estimated to affect nearly five million people worldwide [1,2]. Involvement of the central nervous system (CNS), also known as neuropsychiatric SLE (NPSLE), has a prevalence of up to 45% of SLE patients and is a significant cause of morbidity [3].

NPSLE manifests through a broad range of symptoms, including seizures, psychosis, cognitive impairment, mood disorders, and focal neurological deficits, which create diagnostic and therapeutic challenges due to their diverse presentations [3,4]. The pathophysiology of NPSLE involves multiple mechanisms, such as autoantibody-mediated inflammation, vasculopathy, thrombosis from antiphospholipid syndrome, and blood-brain barrier disruption [3,4]. A key subtype, autoimmune encephalitis, arises when autoantibodies, such as those targeting N-methyl-D-aspartate receptor (NMDAR) subunits or ribosomal P proteins, cause neuronal excitotoxicity and inflammation in limbic regions, leading to seizures, behavioral changes, and cognitive deficits [5].

The absence of specific radiologic or laboratory biomarkers complicates NPSLE diagnosis, requiring a multimodal approach with clinical evaluation, serological testing, and advanced neuroimaging, such as MRI with fluid-attenuated inversion recovery (FLAIR) sequences, to identify underlying mechanisms [3,6]. These distinct pathophysiological subtypes, including vasculitis, ischemic changes, and autoimmune encephalitis, necessitate tailored treatment strategies [3,7,8]. Advanced neuroimaging is critical for distinguishing these mechanisms and guiding effective management of this complex condition [3,4].

## Case presentation

We present a case of a 41-year-old female with a history of SLE. She was first diagnosed with SLE in 2014 with the following clinical features: arthralgias, photosensitivity, Raynaud’s phenomenon, facial discoid lesion, and rashes on the arms, neck, and face. 

In late April of 2022, she presented to the emergency department (ED) with a new onset of generalized convulsive seizure following an episode of unresponsive staring. After two days of hospital admission and Levetiracetam administration (1500 mg IV twice daily), she was discharged. The patient was readmitted to the hospital the next day with more unresponsive staring spells and generalized convulsive seizures. Her medication regimen was then amended to include Valproic acid (500 mg IV twice daily) and lacosamide (150 mg twice daily), in addition to oral prednisone (60 mg daily) for her active lupus. 

On physical examination, the patient was encephalopathic, oriented only to self, with poor speech production, limited fluency, and an inability to follow commands. She responded only to noxious stimuli, stating her name. Cranial nerves were intact without focal deficits, motor strength was 5/5 in all extremities, reflexes were 2+ symmetrically, and there was no pronator drift or dysmetria. Sensory examination was limited due to an altered mental status, but no gross deficits were noted. She later developed neuropsychiatric symptoms, including screaming, agitation, and visual and auditory hallucinations.

A lumbar puncture cerebrospinal fluid (CSF) analysis at this time demonstrated normal composition of the CSF with the exception of mild elevation of protein (81 mg/dL), with glucose level and cell counts within normal range. An electroencephalography (EEG) showed diffuse background slowing with right temporal epileptiform activities. Serology results are summarized in Table 1. Serology results from November 2018 (baseline) are shown beside serology results at the time of her new symptom onset in May 2022 (admission). 

**Table 1:** Relevant serology results throughout clinical course ANA: antinuclear; DNA: deoxyribonucleic acid; AI: antibody index; RNP: ribonucleoprotein; !: Data is abnormal; (H): Data is abnormally high; -: test not performed.

Antibodies	Baseline	Day 1	Reference Range and Units
ANA Qualitative	Positive !	Positive !	Negative
ANA Pattern	Speckled !	-	Reported only if ANA is positive
ANA, Titer	1:1280 !	-	<1:80
Anti-DNA topoisomerase I (SCL-70)	1.0 (H)	0.5	≤0.9 AI
Anti- RNP	>8.0 (H)	>8.0 (H)	≤0.9 AI
Anti-small nuclear ribonucleoprotein (Smith)	0.9	1.3 (H)	≤0.9 AI
Anti-chromatin (nucleosomal)	6.1 (H)	7.5 (H)	≤0.9 AI

**Figure 1:**
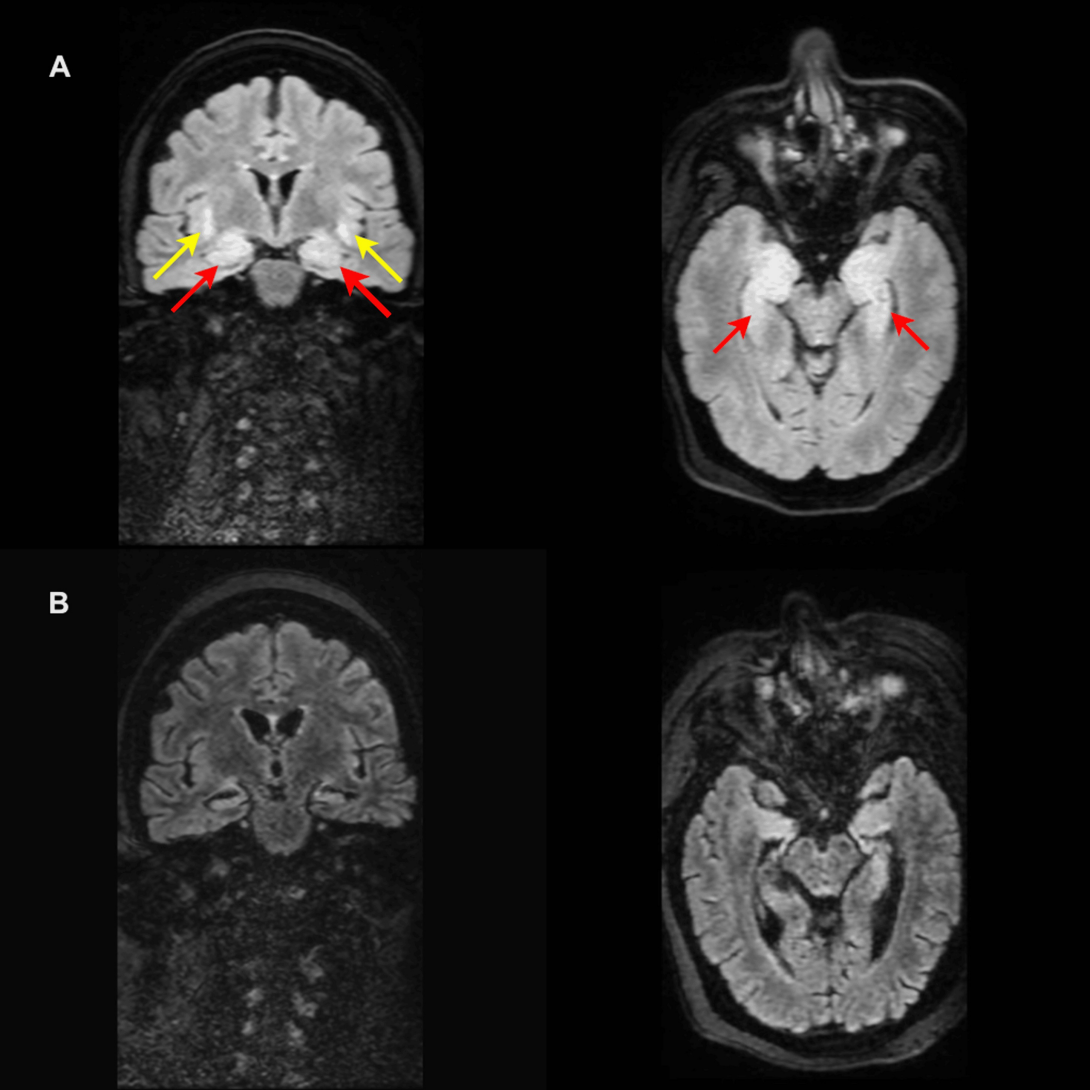
Serial brain MRI FLAIR A shows FLAIR hyperintensities affecting both mesial temporal lobes, including the hippocampi, and the amygdalae with enlargement bilaterally (red arrows), and involving subinsular regions (yellow arrows). B shows significant improvement in high signal intensity and reduction in swelling of FLAIR hyperintensities. FLAIR: fluid-attenuated inversion recovery.

After hospital admission, she developed symptoms of psychosis, including disorganized thought, delusions of persecution, and visual and auditory hallucinations. Levetiracetam was discontinued due to its potential side effects on mental behavior. Despite discontinuation, the patient did not show any clinical improvement and had continued behavioral changes and seizures. The next EEG revealed a focal onset seizure in the right temporal region. MRI showed bilateral medial temporal FLAIR hyperintensities (Figure 1A). Given the MRI findings, IVIg was initiated. She started to have dramatic improvement in her mental status and seizure control two days after IVIg therapy. IV cyclophosphamide (1 g) was then added to her regimen. Overall, the patient’s clinical course began with generalized convulsive seizures on 04/28/2022, progressed to psychosis and behavioral changes by 05/06/2022, and showed dramatic improvement with IVIg initiated on 05/18/2022. At 4-month follow-up, the patient is reported to have “around 1 small seizure per month, which lasts about 15 seconds.” She was able to carry out some activities of daily living, and her cognition was slowly improving. Six months after her hospital MRI in May 2022, repeat imaging showed significant resolution of the FLAIR hyperintensity changes (Figure 1B).

## Discussion

NPSLE may present with a variety of clinical neurological syndromes. Like our patient with serological evidence of active lupus, the CNS involvement may be caused by different pathophysiological mechanisms.

The mechanisms of the clinicopathologic changes in NPSLE may include, but are not limited to, vasculitis, ischemic changes due to antiphospholipid antibody syndrome, blood-brain barrier disruption, or autoimmune encephalitis [3]. Differential diagnoses, including infectious [e.g., Herpes simplex virus (HSV), ruled out by negative CSF polymerase chain reaction (PCR)], paraneoplastic, and other autoimmune encephalitides, must be excluded before attributing encephalitis to SLE [3]. Our patient’s negative computed tomography angiography (CTA) chest/abdomen/pelvis and serological SLE activity supported NPSLE attribution.

Vasculitis, or inflammatory damage to blood vessels, affects nearly 50% of all SLE patients [9]. It can affect any tissue of the body, including the CNS. Patients will present with focal ischemic changes and, more typically, focal neurological findings (i.e., seizures). MRI will show evidence of ischemic infarcts affecting different vascular territories. Our patient did not show evidence of vasculitis-induced ischemic infarcts on MRI [3,10]. In a patient with these findings, treatment is aggressive with steroids and immunosuppressive agents [10].

Another postulated pathophysiological mechanism of NPSLE is ischemic changes due to antiphospholipid (aPL) antibody syndrome. aPL antibodies trigger endothelial cell, platelet, and monocyte activation, which may lead to the formation of small thrombi [11,12]. Furthermore, aPL antibodies expedite atherosclerosis, which is also a known risk factor for cerebrovascular ischemia [11]. Of patients with SLE, aPL antibody-positive individuals are twice as likely to develop focal NPSLE as aPL antibody-negative individuals [3,13]. Our patient does not exhibit brain lesions that are ischemic in nature. For cases with this underlying pathophysiology, treatment typically involves high-dose anticoagulation [5,10,14].

Furthermore, induced disruption of the blood brain barrier (BBB) is relatively common among active SLE patients due to inflammation. This is supported by high levels of cytokines, albumin, and immunoglobulins found in the CSF of NPSLE patients [5]. The infiltration of autoimmune antibodies and other immune mediators into the CNS can lead to neuronal excitotoxicity, demyelination, and inflammation [3]. The patient usually presents with altered mental status and/or seizures. The MRI will show evidence of cerebral edema, which is not present in our case. The treatment in this case is high-dose corticosteroids [5,10,14].

Lastly, autoimmune encephalitis is the most rare pathophysiological subtype of NPSLE. While the mechanism is not completely understood, it is thought that invasion of antibodies into the BBB and choroid plexus may result in neuronal excitotoxicity [3]. In SLE-associated limbic encephalitis, autoantibodies such as anti-NR2A/B or anti-ribosomal P target neuronal antigens, causing inflammation and synaptic dysfunction [5]. Our patient’s elevated anti-ribonucleoprotein (RNP) [>8.0 antibody index (AI)] and anti-chromatin (7.5 AI) suggest systemic inflammation amplifying blood-brain barrier permeability [5]. Her presentation with bilateral limbic system encephalitis, including behavioral changes, temporal lobe seizures, and MRI findings of FLAIR high signal areas and enlargement in both amygdalae, suggests nonspecific inflammation of the medial temporal lobe, supporting this diagnosis. The patient’s dramatic improvement within 48 hours of IVIg, with restored ability to converse and use her cell phone, underscores its therapeutic value in antibody-mediated NPSLE. 

Diagnosing SLE-associated limbic encephalitis requires a multimodal approach, including MRI, EEG, CSF analysis, serology, and ideally antibody panels to rule out other causes [e.g., NMDAR, α-amino-3-hydroxy-5-methyl-4-isoxazolepropionic acid receptor (AMPAR) antibodies] [15-17]. Our case included MRI (bilateral temporal hyperintensities), EEG (right temporal seizures), CSF (elevated protein 81 mg/dL, normal cells/glucose, negative viral panel), and serology [ANA: antinuclear (ANA) 1:1280, anti-RNP >8.0 AI], but testing for anti-NMDA antibodies and other anti-neurological antibodies was not conducted due to resource limitations. Oligoclonal bands were normal on 05/04/2022, but the IgG index was not measured. Negative CTA and CSF viral panel, alongside clinical and imaging findings, align with cases like Zaeem et al. (2020) and Kano et al. (2009), supporting NPSLE attribution [16,17]. This highlights the need for comprehensive panels in future diagnostics to refine attribution.

## Conclusions

This case highlights the value of integrating clinical presentation with neuroimaging to elucidate the probable pathophysiology of NPSLE. Our patient’s bilateral medial temporal FLAIR hyperintensities and rapid response to intravenous immunoglobulin strongly suggest an autoimmune limbic encephalitis subtype of NPSLE. Recognizing these imaging features can guide timely, targeted interventions when conventional treatment fails. As NPSLE encompasses a spectrum of mechanisms, from vasculitis to antibody-mediated neuroinflammation, a better understanding of these subtypes is essential for optimizing patient outcomes. Advanced MRI techniques offer a non-invasive means to support diagnosis and inform treatment decisions in this challenging and varied condition.

## References

[1] (2025). Lupus facts and statistics. https://www.lupus.org/resources/lupus-facts-and-statistics.

[2] Klippel JH (1997). Systemic lupus erythematosus: demographics, prognosis, and outcome. J Rheumatol Suppl.

[3] Ota Y, Srinivasan A, Capizzano AA (2022). Central nervous system systemic lupus erythematosus: pathophysiologic, clinical, and imaging features. Radiographics.

[4] Rice-Canetto TE, Joshi SJ, Kyan KA, Siddiqi J (2024). Neuropsychiatric systemic lupus erythematosus: a systematic review. Cureus.

[5] Schwartz N, Stock AD, Putterman C (2019). Neuropsychiatric lupus: new mechanistic insights and future treatment directions. Nat Rev Rheumatol.

[6] Vivaldo JF, de Amorim JC, Julio PR, de Oliveira RJ, Appenzeller S (2018). Definition of NPSLE: does the ACR nomenclature still hold?. Front Med (Lausanne).

[7] Gegenava M, Beaart HJ, Monahan RC (2019). Performance of the proposed ACR-EULAR classification criteria for systemic lupus erythematosus (SLE) in a cohort of patients with SLE with neuropsychiatric symptoms. RMD Open.

[8] Muscal E, Brey RL (2010). Neurologic manifestations of systemic lupus erythematosus in children and adults. Neurol Clin.

[9] Leone P, Prete M, Malerba E, Bray A, Susca N, Ingravallo G, Racanelli V (2021). Lupus vasculitis: an overview. Biomedicines.

[10] Fanouriakis A, Tziolos N, Bertsias G, Boumpas DT (2021). Update οn the diagnosis and management of systemic lupus erythematosus. Ann Rheum Dis.

[11] Cohen D, Rijnink EC, Nabuurs RJ (2017). Brain histopathology in patients with systemic lupus erythematosus: identification of lesions associated with clinical neuropsychiatric lupus syndromes and the role of complement. Rheumatology (Oxford).

[12] Schreiber K, Sciascia S, de Groot PG (2018). Antiphospholipid syndrome. Nat Rev Dis Primers.

[13] Ho RC, Thiaghu C, Ong H, Lu Y, Ho CS, Tam WW, Zhang MW (2016). A meta-analysis of serum and cerebrospinal fluid autoantibodies in neuropsychiatric systemic lupus erythematosus. Autoimmun Rev.

[14] Sarwar S, Mohamed AS, Rogers S (2021). Neuropsychiatric systemic lupus erythematosus: a 2021 update on diagnosis, management, and current challenges. Cureus.

[15] Emerson JS, Gruenewald SM, Gomes L, Lin MW, Swaminathan S (2023). The conundrum of neuropsychiatric systemic lupus erythematosus: current and novel approaches to diagnosis. Front Neurol.

[16] Kano O, Arasaki K, Ikeda K, Aoyagi J, Shiraishi H, Motomura M, Iwasaki Y (2009). Limbic encephalitis associated with systemic lupus erythematosus. Lupus.

[17] Zaeem Z, Luk CC, Anderson D, Blevins G, Siddiqi ZA (2020). AMPA-R limbic encephalitis associated with systemic lupus erythematosus. Can J Neurol Sci.

